# A case report of an STEMI mimicker in a patient presenting with haemoptysis and chest pain with metastatic myocardial infiltration and left ventricular mural thrombi

**DOI:** 10.1093/ehjcr/ytaa546

**Published:** 2021-01-05

**Authors:** Ruihai Zhou, Sudhir Prasada, Michael Roth

**Affiliations:** 1 Division of Cardiology, Department of Medicine, University of North Carolina at Chapel Hill, Chapel Hill, NC, USA; 2 Department of Pathology, Nash General Hospital of University of North Carolina Health Care

**Keywords:** Case report, ST-elevation myocardial infarction, Coronary angiography, Cardiac metastasis, Ventricular mural thrombus, Echocardiography, Cardiac magnetic resonance, Positron emission tomography

## Abstract

**Background:**

ST-elevation myocardial infarction (STEMI) requires timely coronary reperfusion but localizing ST-segment elevation (STE) can develop in clinical settings other than STEMI.

**Case summary:**

We report a case of a 66-year-old man, with a history of diabetes mellitus and arthritis presenting with haemoptysis and chest pain. The electrocardiogram (ECG) at presentation showed marked localizing STE but emergent cardiac catheterization showed no significant coronary artery obstruction and the serial serum cardiac troponin levels were within normal limits. The patient was found to have squamous cell carcinoma with a right upper lobe cavitated lung mass and cardiac infiltrative metastasis as shown by computed tomography, echocardiography, cardiac magnetic resonance, and 18F-fluorodeoxyglucose*-*positron emission tomography-computed tomography (FDG-PET-CT) imaging. Mobile left ventricular mural thrombi were also noted on echocardiography.

**Discussion:**

Metastatic myocardial infiltration can cause STE mimicking STEMI on ECG. The STE is persistent and may reflect an ongoing injury current between the infiltrated and normal myocardium. The STE is localizing, which may have value in evaluating the extent and region of metastatic myocardial damage. Myocardial metastasis can be complicated by ventricular mural thrombosis and due to lack of population data, there is no firm guidance on choice of anticoagulation.

Learning points

- Metastatic myocardial infiltration can cause persistent ST-segment elevation (STE) on electrocardiogram with normal cardiac troponin level.
- The STE is localizing and could be used to estimate the extent of metastatic myocardial damage.
- Metastatic myocardial infiltration can cause cardiac mural thrombosis, and due to lack of population data, there is no firm guidance on choice of anticoagulation.

## Introduction

ST-elevation myocardial infarction (STEMI) is diagnosed in the setting of characteristic symptoms of myocardial ischaemia in association with persistent ST-segment elevation (STE) on electrocardiogram (ECG) and subsequent release of biomarkers of myocardial necrosis. Diagnostic STE in the absence of left ventricular hypertrophy or left bundle-branch block is defined by the European Society of Cardiology/ACCF/AHA/World Heart Federation Task Force for the Universal Definition of Myocardial Infarction as new STE of ≥0.1 mV at the J point in 2 contiguous leads in all leads other than leads V2–V3, or in V2–V3 of ≥0.2 mV in men ≥40years, ≥0.25 mV in men <40 years, or ≥0.15 mV in women. ST-elevation myocardial infarction requires timely coronary revascularization. We report a case of ‘supposed STEMI’ presenting with marked STE on ECG but cardiac catheterization showed no significant coronary obstruction, in the setting of newly diagnosed lung cancer with myocardial metastasis.

## Timeline

**Table:** 

Day 0	A 66-year-old man presented with haemoptysis and chest pain with electrocardiogram showing marked localizing STE in multiple leads triggering a STEMI code.
Coronary angiography showed no significant coronary obstruction. Chest computed tomography revealed a large cavitated lung mass within the right upper lobe. Transthoracic echocardiography showed heterogeneously echogenic left ventricular focal wall thickening with regional motion abnormality and mural thromibi.	
Day 3	Needle biopsy of a right deltoid muscle mass showed metastatic squamous cell carcinoma.
Day 10	18F-fluorodeoxyglucose*-*positron emission tomography-computed tomography (FDG-PET-CT) imaging showed hypermetabolic myocardial infiltration and mass in the right upper lobe.
Day 25	Cardiac magnetic resonance imaging showed metastatic myocardial infiltration in regions well reflected by electrocardiographic STE localization.
Day 41	Palliative chemoimmunotherapy was started.
Day 59	The STE of same amplitude and location was still present on the follow-up ECG obtained 2 months after clinical presentation and the patient has been clinically stable from a cardiac standpoint.

## Case presentation

A 66-year-old male with a history of type 2 diabetes mellitus and osteoarthritis, on no prescribed medications, presented with haemoptysis and chest pain. On examination, the temperature was 36.7°C, the blood pressure 84/69 mmHg, the heart rate 130 b.p.m., the respiratory rate 18 breaths per minute, and the oxygen saturation 100% on room air. Cardiopulmonary exam was overall unremarkable. A mass was noted in the right deltoid muscle region.

**Figure 1:**
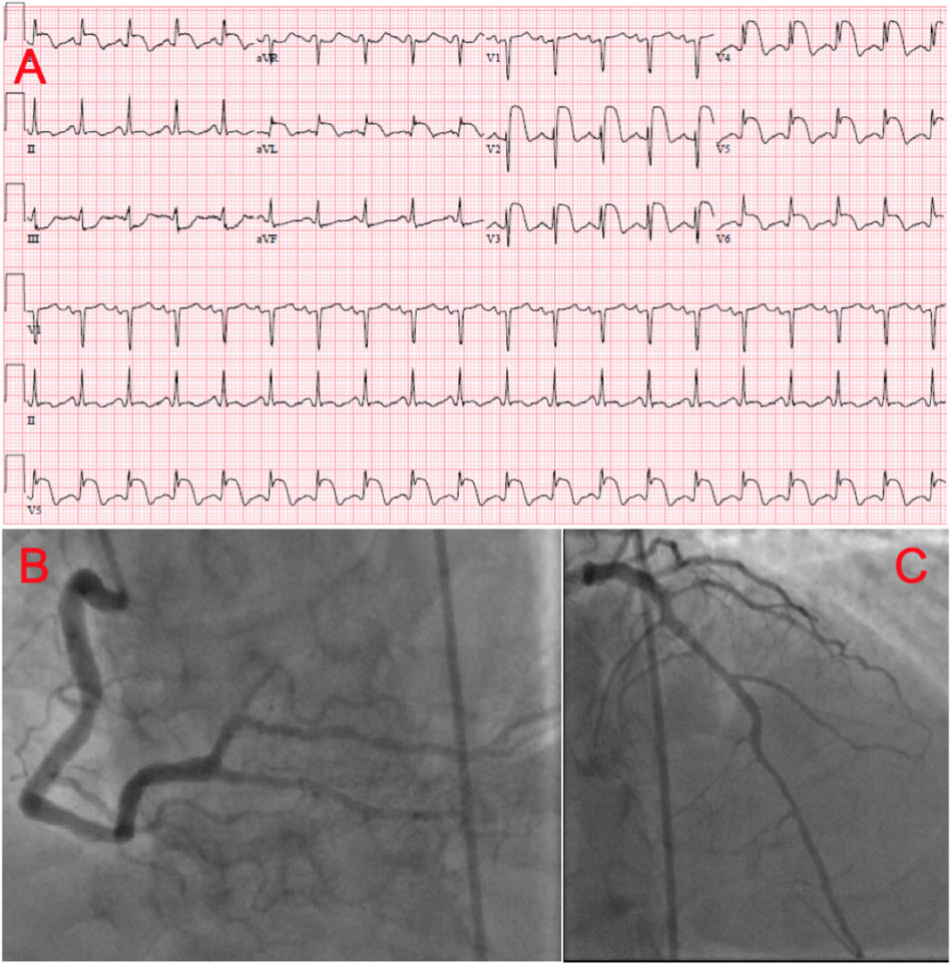
(*A*) Electrocardiogram at presentation showing ST-segment elevation in multiple leads. (*B*, *C*) Coronary angiography showing non-obstructive coronary artery disease. (*B*) Right and (*C*) left coronary arteries.

The ECG at presentation showed sinus tachycardia with a heart rate of 117 b.p.m. and marked convex STE in multiple leads including V2–V6, I, and aVL, with reciprocal changes (*Figure 1*). ST-elevation myocardial infarction code was activated. Emergent coronary angiography showed no significant coronary obstruction (*Figure 1*). The STE was persistent on subsequent ECGs and blood cardiac troponin I (cTnI) levels were within normal range (<0.3 ng/mL). The complete blood count and basic metabolic panel were unremarkable except for mild hyponatraemia with sodium level of 130 mEq/L (normal sodium level 135–145 mEq/L) and mildly elevated fasting plasma glucose level of 7.1 mmol/L. The chest X-ray showed a right upper lobe wedge-shaped airspace consolidation.

**Figure 2:**
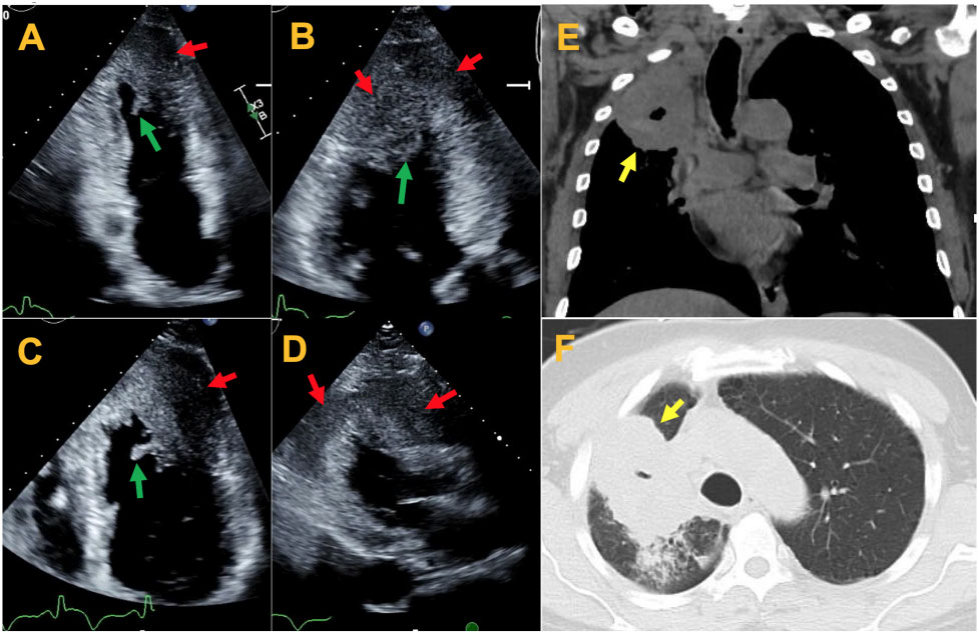
(*A–D*) Transthoracic echocardiography showing thickened wall in different views with heterogenous echogenicity (red arrows) and mobile left ventricular mural thrombi (green arrows). (*E*, *F*) Computed tomography (CT) imaging showing cavitated right upper lobe mass (yellow arrows).

**Figure 3:**
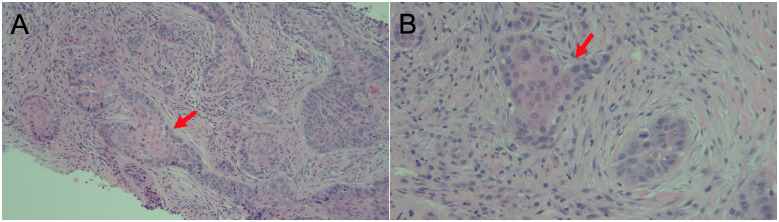
Histological analysis of the biopsied mass in the right deltoid muscle showing squamous cell carcinoma with infiltrating nests (red arrow) of moderately differentiated, keratinizing, malignant squamous cells with a desmoplastic background replacing skeletal muscle. Original magnification, *A* ×100, *B* ×200.

The transthoracic echocardiogram (TTE) showed severe focal wall thickening and hypokinesis/akinesis in apical, apical lateral, anteroseptal, and anterior wall. The thickened wall is characterized by heterogenous echogenicity different from that of normal myocardium. Mural mobile echodensities associated with the hypokinetic/akinetic wall were seen in the left ventricle, consistent with mural thrombi (*Figure 2A–D*, *Video 1*). Computed tomography (CT) of the chest (*Figure 2E* and *F*) showed a large partially cavitated lung mass within the right upper lobe. Given the haemoptysis and cavitated lung mass, thus bleeding concern, anticoagulation was held. Ultrasound-guided needle biopsy of the right deltoid muscle mass was performed, and the histological analysis showed infiltrating squamous cell carcinoma (*Figure 3*).

**Figure 4:**
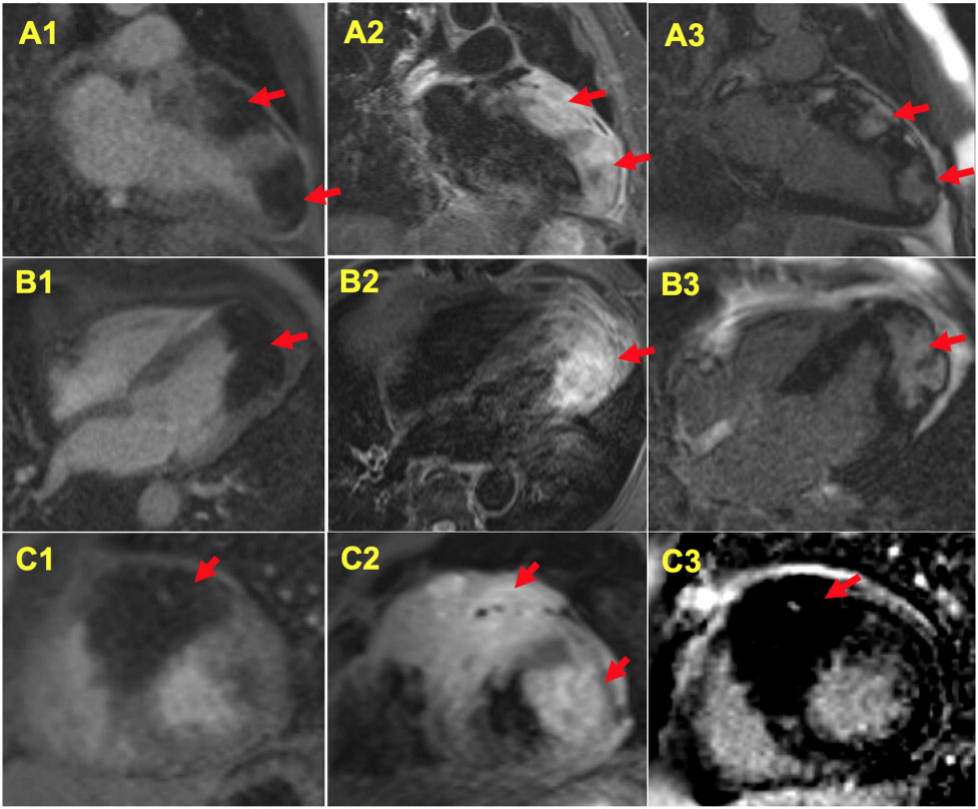
Cardiac magnetic resonance (CMR) imaging using different pulse sequences in two-chamber (A1–A3), four-chamber (B1–B3), and short axis (C1–C3) views. Infiltrative masses involving the apical, apical lateral, apical-mid anteroseptal, and anterior wall are noted (arrows). (*A1*, *B1*, *C1*) On first-pass perfusion imaging, the cardiac masses had poor perfusion indicating either no coronary artery perfusion, avascular, or necrosis. (*A2*, *B2*, *C2*) T2 short-tau inversion recovery (STIR) imaging showed increased signals in the infiltrated mass with heterogeneous intensity. (*A3*, *B3*, *C3*) Imaging using phase-sensitive inversion recovery (PSIR) sequences showed patchy delayed hyperenhancement in the infiltrative masses.

**Figure 5:**
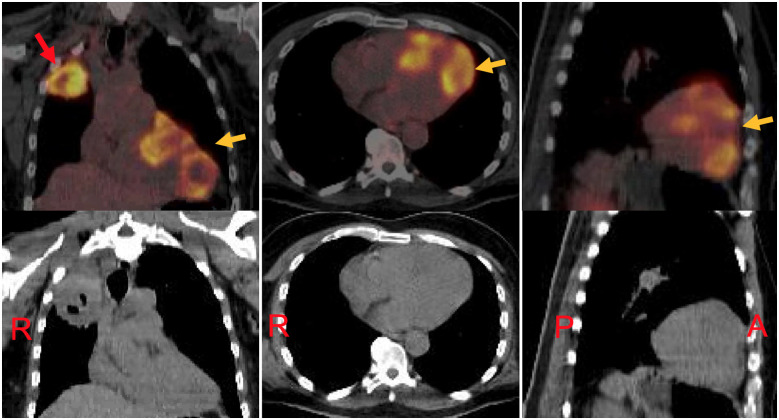
An 18F-fluorodeoxyglucose*-*positron emission tomography-computed tomography (FDG-PET-CT) imaging showing hypermetabolic masses consistent with myocardial metastases (orange arrows) and hypermetabolic mass within right upper lung lobe consistent with primary malignancy (red arrow). Computed tomography images are shown in lower panel. A, anterior; P, posterior; R, right.

In further delineating the myocardial damage, cardiac magnetic resonance (CMR) was performed, which showed focal wall thickening with regional hypokinesis/akinesis due to infiltrative masses involving apical, apical lateral, anteroseptal, and anterior wall, consistent with malignant cardiac metastases (*Figure 4*, *Video 2*). An 18F-fluorodeoxyglucose (FDG)*-*positron emission tomography (PET)-CT imaging showed large areas of hypermetabolic activity corresponding to the myocardial metastasis as shown on CMR imaging and a large hypermetabolic mass in the right upper lobe consistent with primary lung malignancy (*Figure 5*).

The patient was seen by oncology and diagnosed with stage IV lung squamous cell carcinoma. Palliative chemoimmunotherapy was started. The persistent STE was still present on the follow-up ECG obtained 2 months after clinical presentation and the patient remained clinically stable from a cardiac standpoint.

## Discussion

We present a case of a patient presenting with haemoptysis and chest pain with marked STE triggering an STEMI code but the emergent cardiac catheterization showed no coronary obstruction. The patient was found to have metastatic squamous cell lung cancer and malignant myocardial infiltration.

STEMI is by definition caused by coronary occlusion. If not treated in a timely fashion, subsequent necrosis of myocardium will occur, and the STE is secondary to the injury current between the normal and ischaemic myocardia. In our case, the mechanism underlying the persistent STE is unclear. The TTE showed regional thickened myocardium, different in echogenicity from the surrounding normal appearing myocardium. Cardiac magnetic resonance imaging showed poor blood perfusion and heterogeneous signal intensities on the short-tau inversion recovery (STIR) and phase-sensitive inversion recovery (PSIR) sequences in the infiltrating mass, indicating partial necrosis and oedema. The difference in cellularity and cell bioelectrical property between the infiltrating neoplasm and normal myocardium may create electropotential difference and resultant ‘injury current’ formation causing STE.^1^^,^^2^ Surprisingly, serial levels of cTnI on three occasions were not elevated in our case, which further ruled out STEMI.

The STE on ECG showed a localizing pattern with reciprocal ST changes reflecting the infiltrated myocardial territories as revealed by the TTE, CMR, and PET-CT imaging. Using the keywords ‘ST elevation’ and ‘cardiac metastasis’ for PubMed search, up to the day of manuscript submission, there are 28 published reports of STE in the patients with myocardial metastasis from malignancies.^3^ Review of those reports with description of or imaging data showing the extent of myocardial involvement revealed that the STE was localized to the myocardial territory involved. Therefore, the STE on ECG in the patients with cardiac metastasis has value in assessing the extent and region of myocardial damage.

Mural mobile echodensities were noted in the left ventricle on TTE indicating mural thrombi. Endocardial endothelial denudation or dysfunction can create a substrate for mural thrombosis. Population data on anticoagulation for cardiac metastasis-associated mural thrombosis is lacking. Therefore if anticoagulant is used, warfarin vs. non-vitamin K oral anticoagulant (NOAC) selection is empiric, although off-label use of NOACs is less effective than coumadin in preventing thromboembolism from left ventricular thrombi in the setting of various cardiomyopathies.^4^

## Lead author biography

**Figure:**
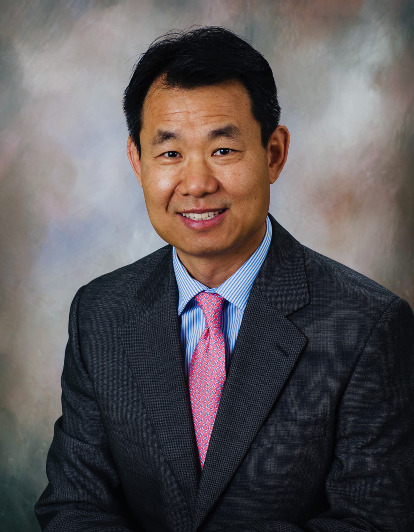


Ruihai Zhou, MSc, MD, FACC, RPVI, is a cardiologist on the faculty of Division of Cardiology, Department of Medicine, University of North Carolina at Chapel Hill, USA. He is board certified by American Board of Internal Medicine (ABIM) in internal medicine, cardiovascular disease, and interventional cardiology. Dr Zhou is also a registered physician in vascular interpretation (RPVI) certified by the Alliance for Certification & Advancement (APCA). He has training and basic research experience in pharmacology, clinical pharmacology, and molecular and cell biology, in addition to his clinical training.

## Supplementary material

Supplementary material is available at *European Heart Journal - Case Reports* online.

**Slide sets:** A fully edited slide set detailing this case and suitable for local presentation is available online as Supplementary data.

**Consent:** The authors confirm that consent for submission and publication of this case report including images and associated text has been obtained from the patient in line with COPE guidance.

**Conflict of interest:** none declared.

**Funding:** none declared.

## Supplementary Material

ytaa546_Supplementary_Data
