# Supplementary material for: A case report of an STEMI mimicker in a patient presenting with haemoptysis and chest pain with metastatic myocardial infiltration and left ventricular mural thrombi
Source: Eur Heart J Case Rep. 2021 Jan 5;5(2):ytaa546. doi: 10.1093/ehjcr/ytaa546 (PMC7954242; doi:10.1093/ehjcr/ytaa546)
Supplement: ytaa546_Supplementary_Data [file ytaa546_Supplementary_Data.zip › EHJ-CR-Slide-Set-EHJ-CR-D-20-01000 ZHOU.pptx]

## Slide 1
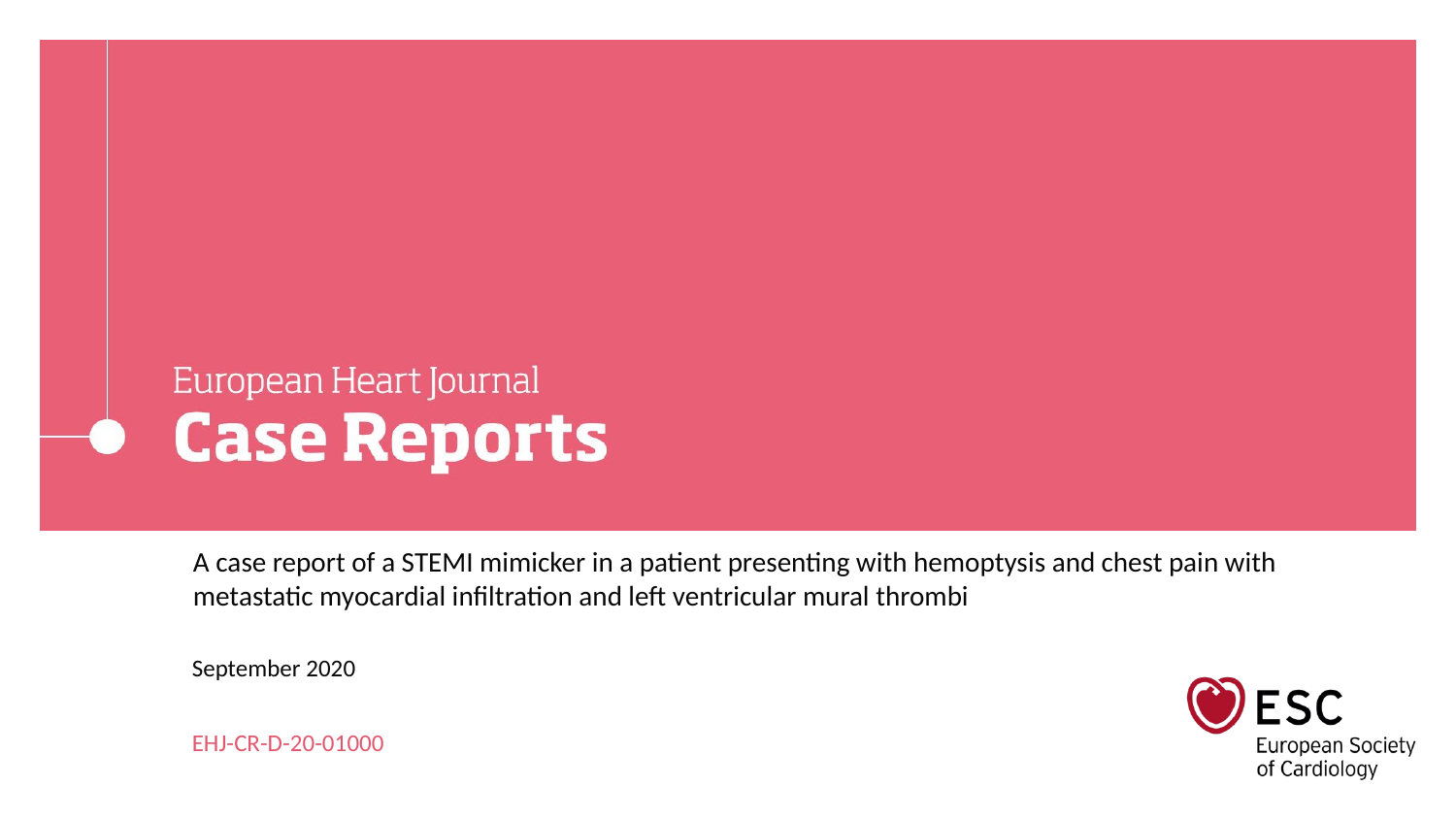

# A case report of a STEMI mimicker in a patient presenting with hemoptysis and chest pain with metastatic myocardial infiltration and left ventricular mural thrombi
September 2020
EHJ-CR-D-20-01000

## Slide 2
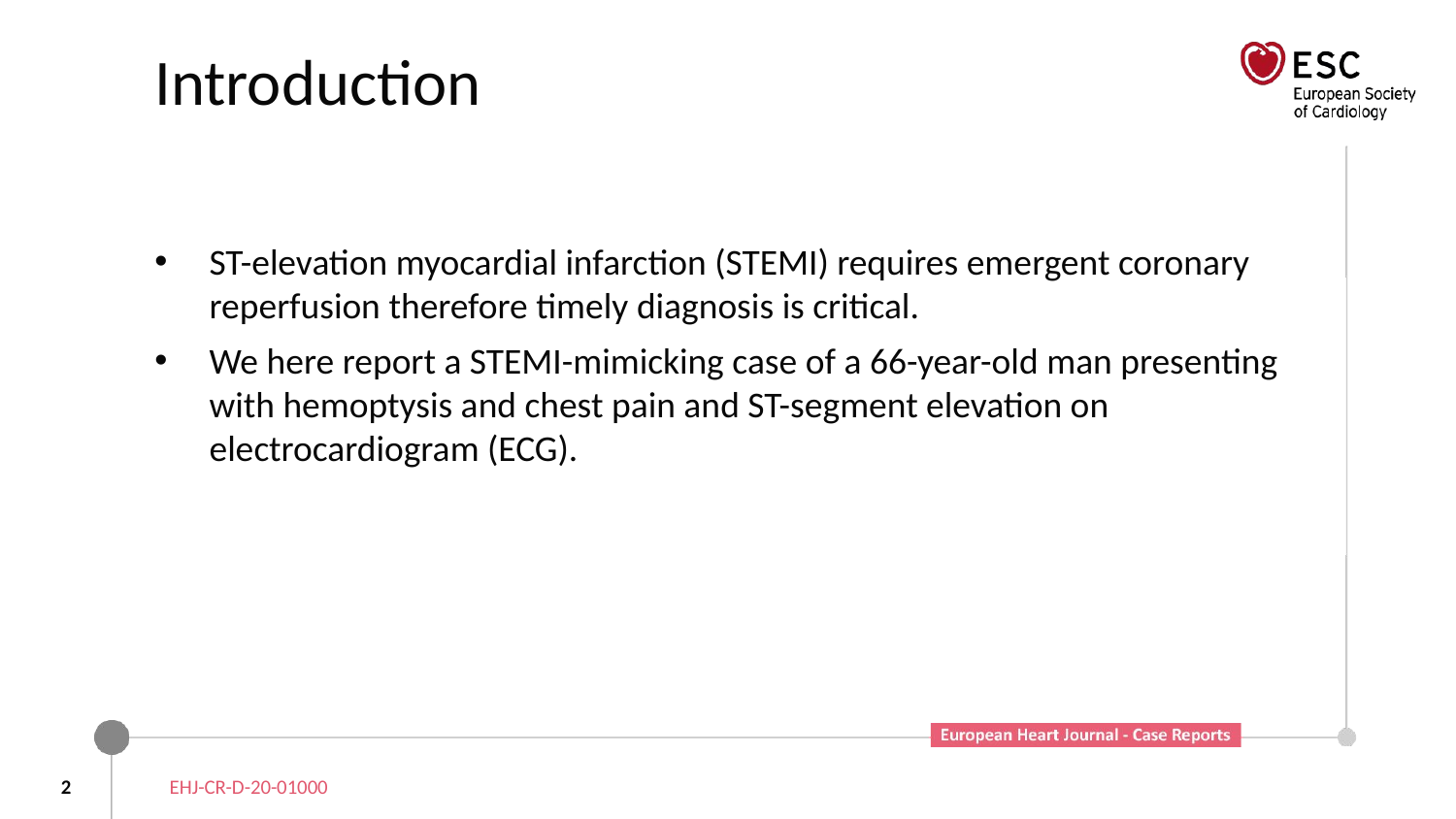

# Introduction
ST-elevation myocardial infarction (STEMI) requires emergent coronary reperfusion therefore timely diagnosis is critical.
We here report a STEMI-mimicking case of a 66-year-old man presenting with hemoptysis and chest pain and ST-segment elevation on electrocardiogram (ECG).
2
EHJ-CR-D-20-01000

## Slide 3
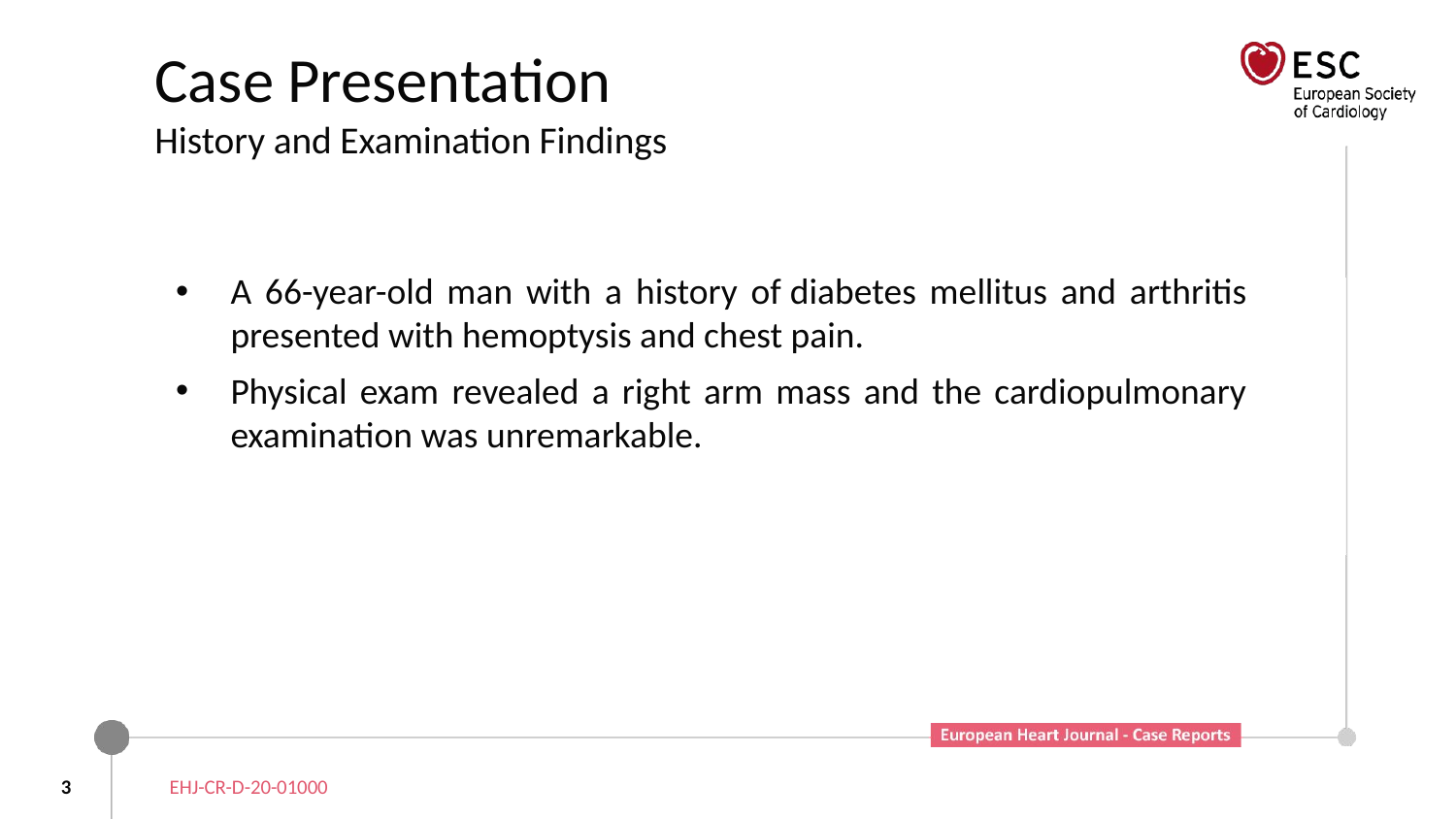

# Case Presentation History and Examination Findings
A 66-year-old man with a history of diabetes mellitus and arthritis presented with hemoptysis and chest pain.
Physical exam revealed a right arm mass and the cardiopulmonary examination was unremarkable.
3
EHJ-CR-D-20-01000

## Slide 4
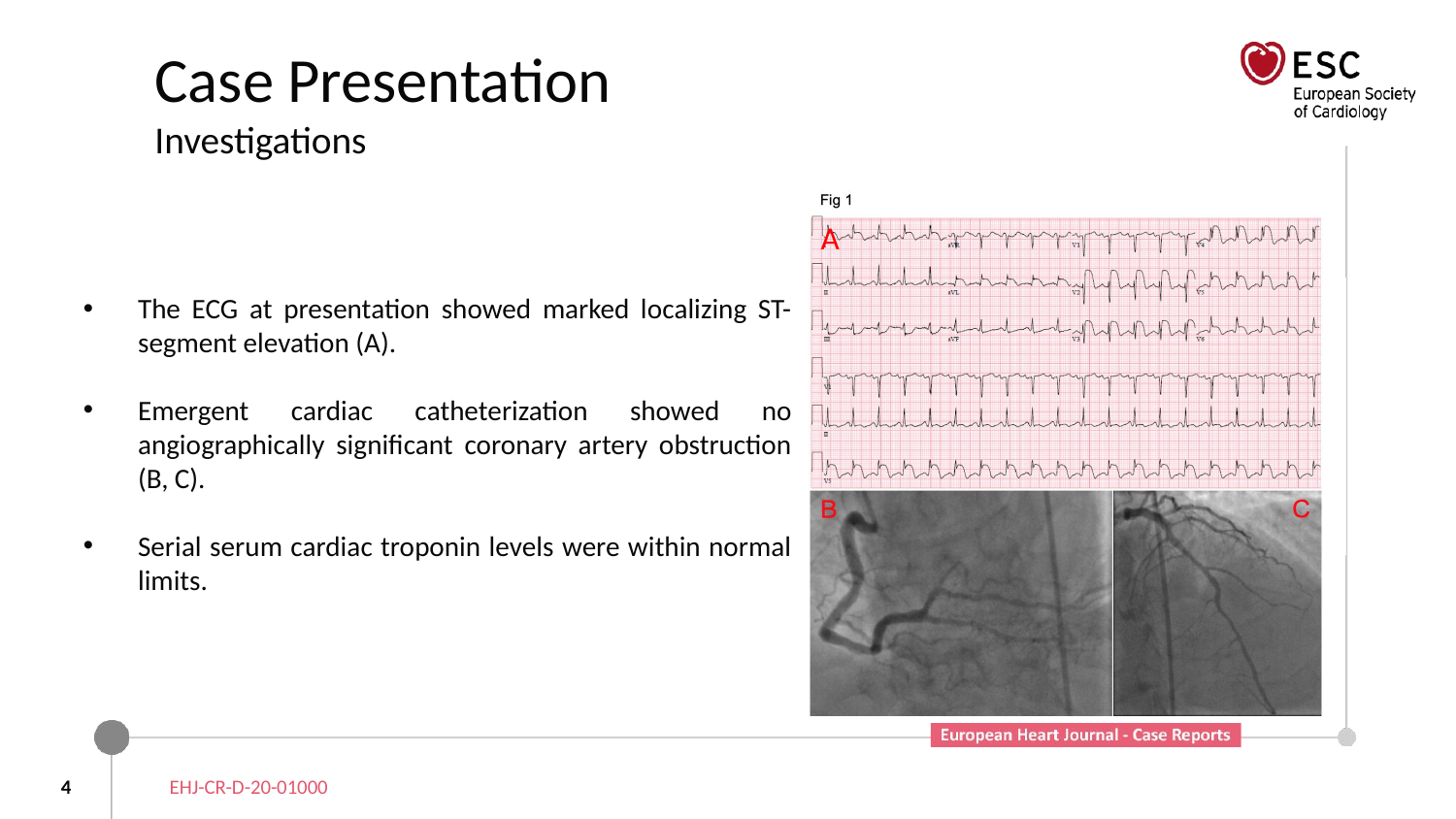

# Case Presentation Investigations
A
The ECG at presentation showed marked localizing ST-segment elevation (A).
Emergent cardiac catheterization showed no angiographically significant coronary artery obstruction (B, C).
Serial serum cardiac troponin levels were within normal limits.
4
EHJ-CR-D-20-01000

## Slide 5
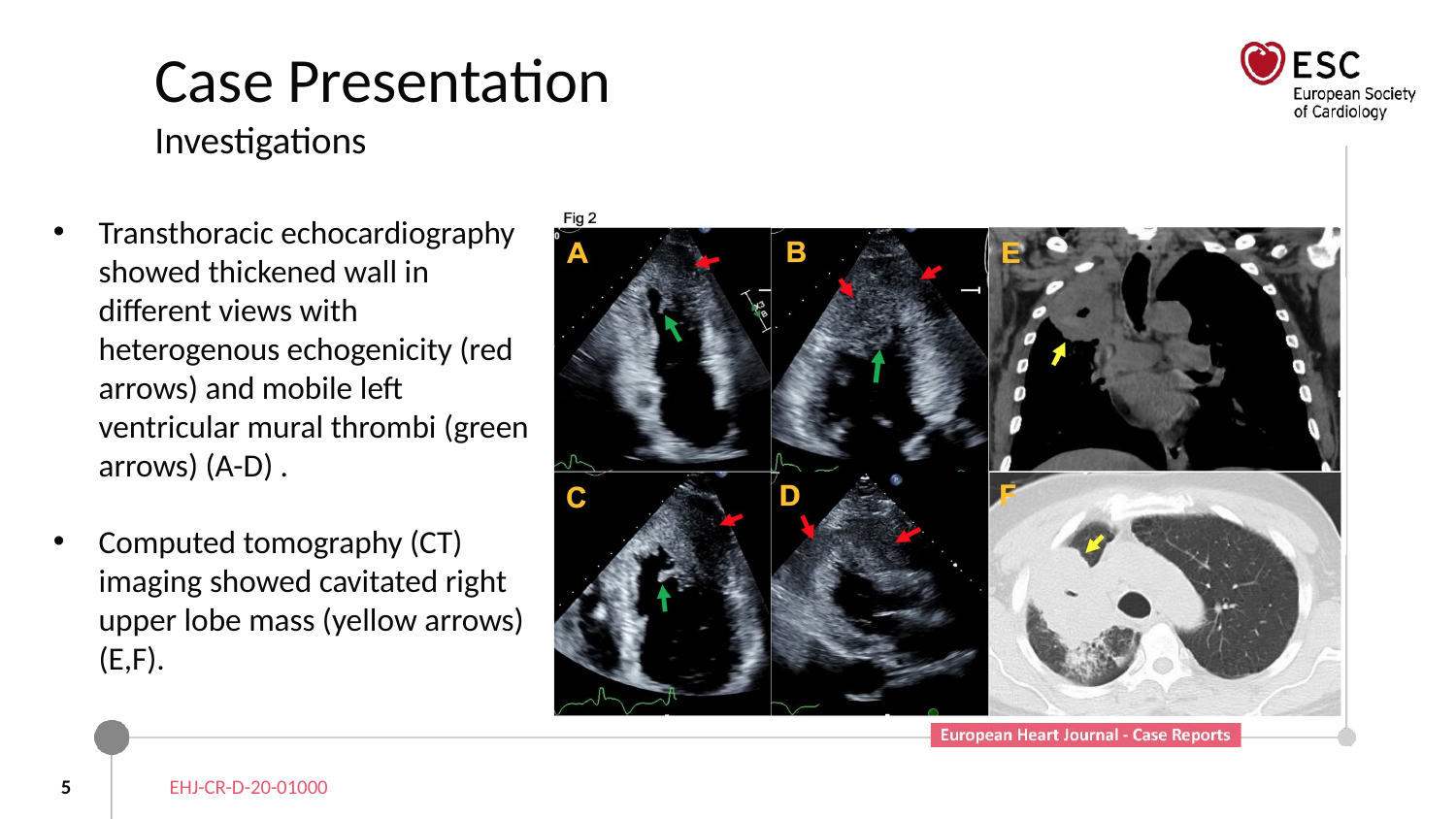

# Case Presentation Investigations
Transthoracic echocardiography showed thickened wall in different views with heterogenous echogenicity (red arrows) and mobile left ventricular mural thrombi (green arrows) (A-D) .
Computed tomography (CT) imaging showed cavitated right upper lobe mass (yellow arrows) (E,F).
5
EHJ-CR-D-20-01000

## Slide 6
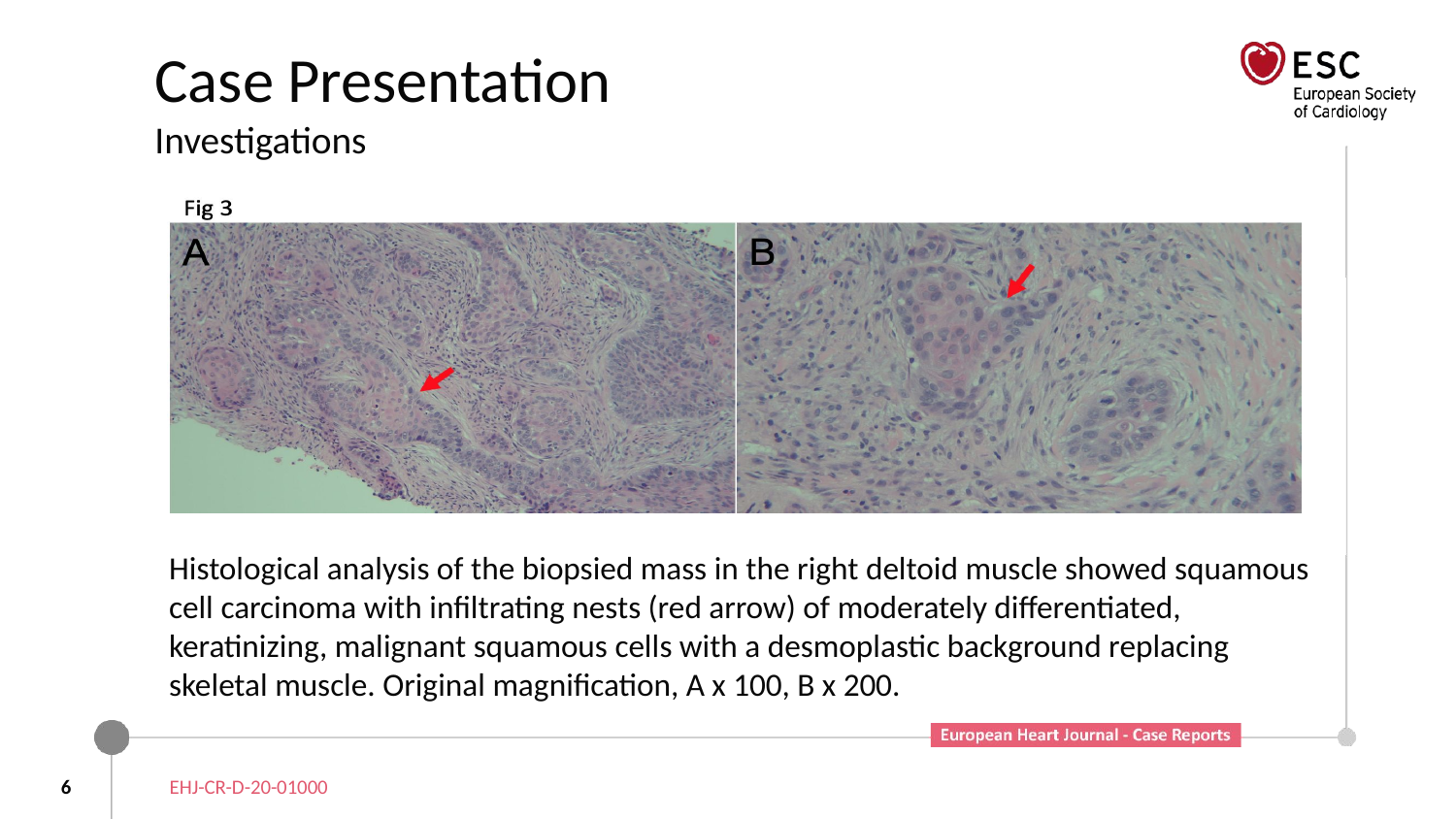

# Case Presentation Investigations
Histological analysis of the biopsied mass in the right deltoid muscle showed squamous cell carcinoma with infiltrating nests (red arrow) of moderately differentiated, keratinizing, malignant squamous cells with a desmoplastic background replacing skeletal muscle. Original magnification, A x 100, B x 200.
6
EHJ-CR-D-20-01000

## Slide 7
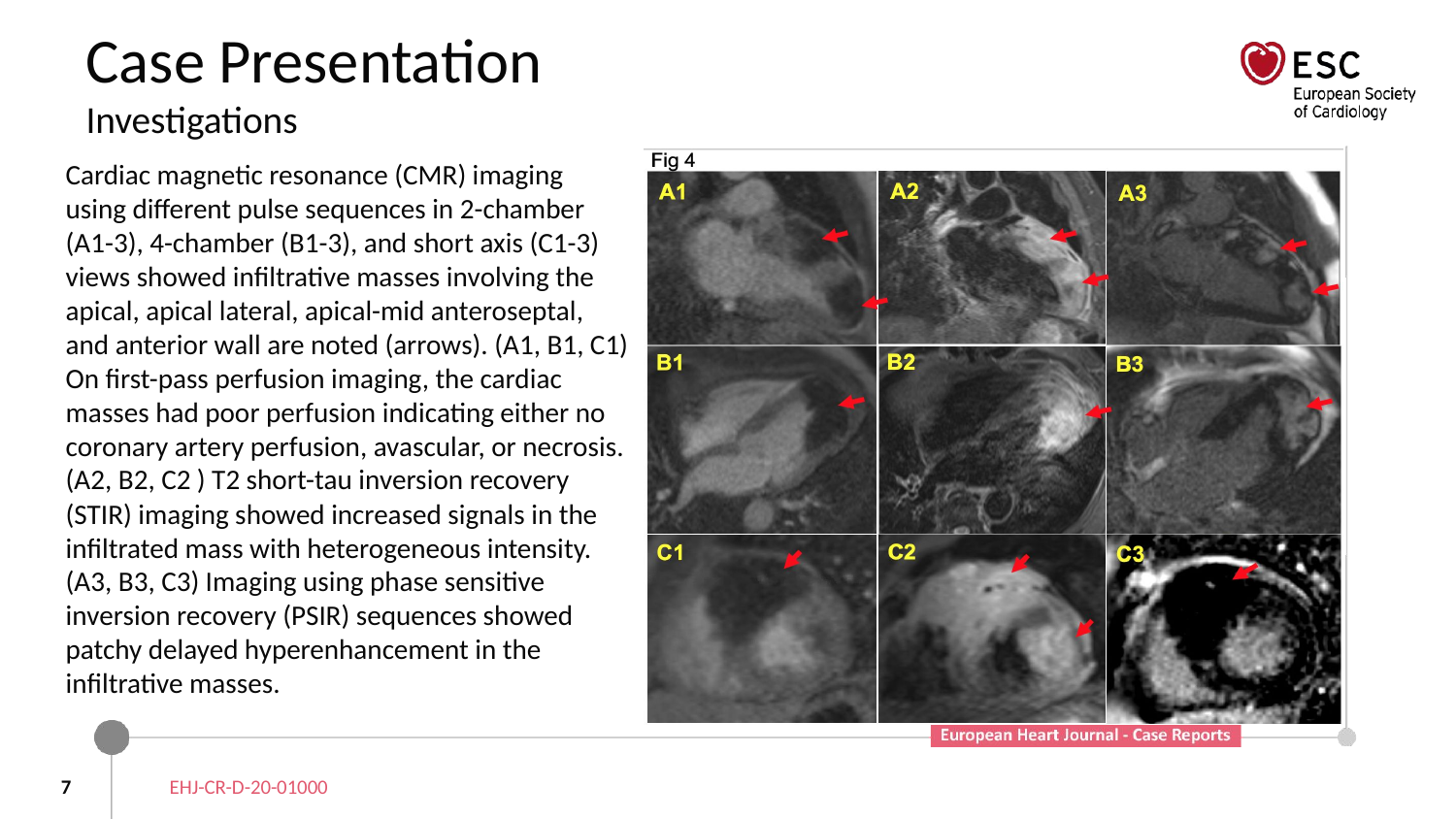

# Case Presentation Investigations
Cardiac magnetic resonance (CMR) imaging using different pulse sequences in 2-chamber (A1-3), 4-chamber (B1-3), and short axis (C1-3) views showed infiltrative masses involving the apical, apical lateral, apical-mid anteroseptal, and anterior wall are noted (arrows). (A1, B1, C1) On first-pass perfusion imaging, the cardiac masses had poor perfusion indicating either no coronary artery perfusion, avascular, or necrosis. (A2, B2, C2 ) T2 short-tau inversion recovery (STIR) imaging showed increased signals in the infiltrated mass with heterogeneous intensity. (A3, B3, C3) Imaging using phase sensitive inversion recovery (PSIR) sequences showed patchy delayed hyperenhancement in the infiltrative masses.
7
EHJ-CR-D-20-01000

## Slide 8
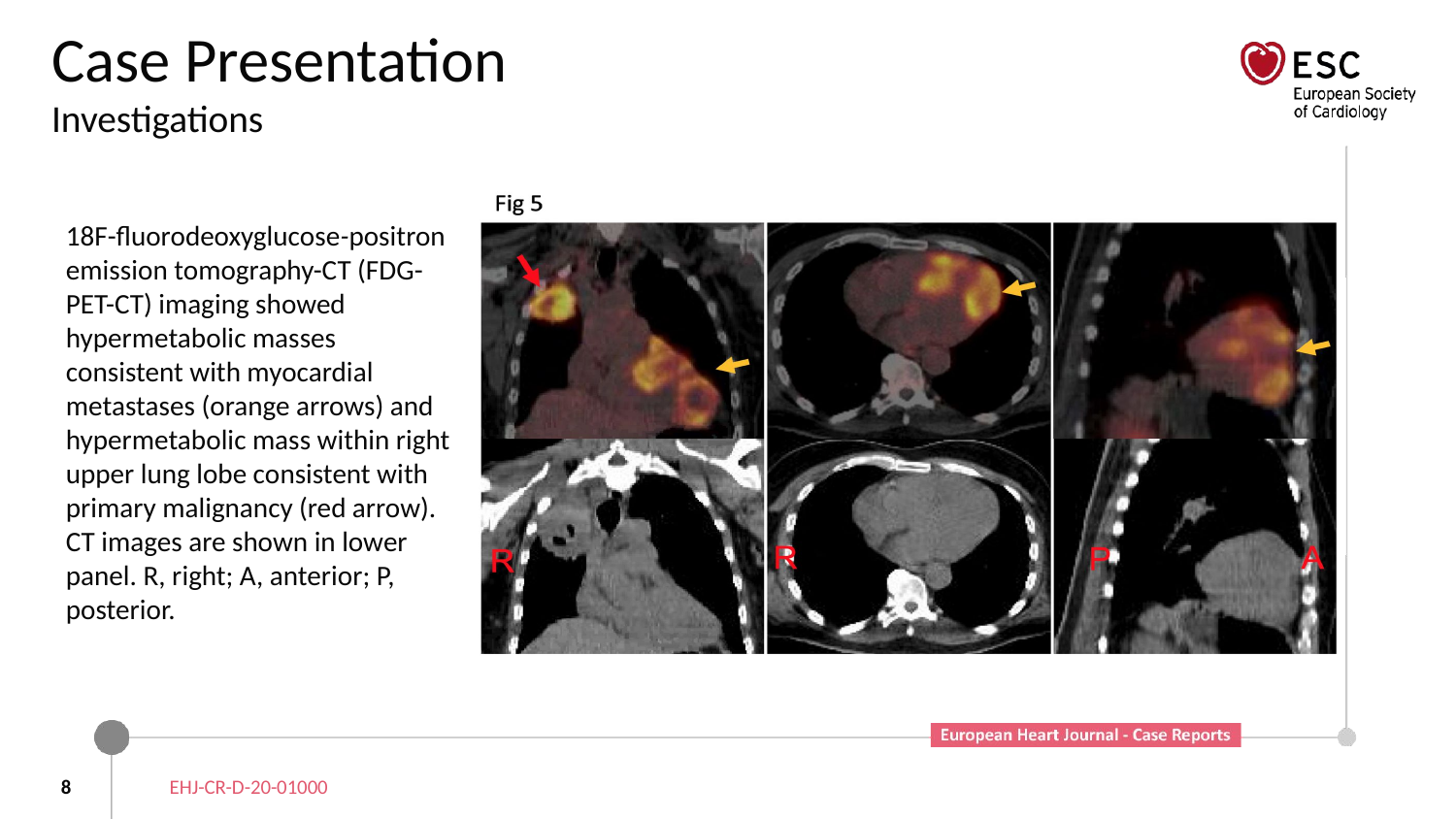

# Case Presentation Investigations
18F-fluorodeoxyglucose-positron emission tomography-CT (FDG-PET-CT) imaging showed hypermetabolic masses consistent with myocardial metastases (orange arrows) and hypermetabolic mass within right upper lung lobe consistent with primary malignancy (red arrow). CT images are shown in lower panel. R, right; A, anterior; P, posterior.
8
EHJ-CR-D-20-01000

## Slide 9
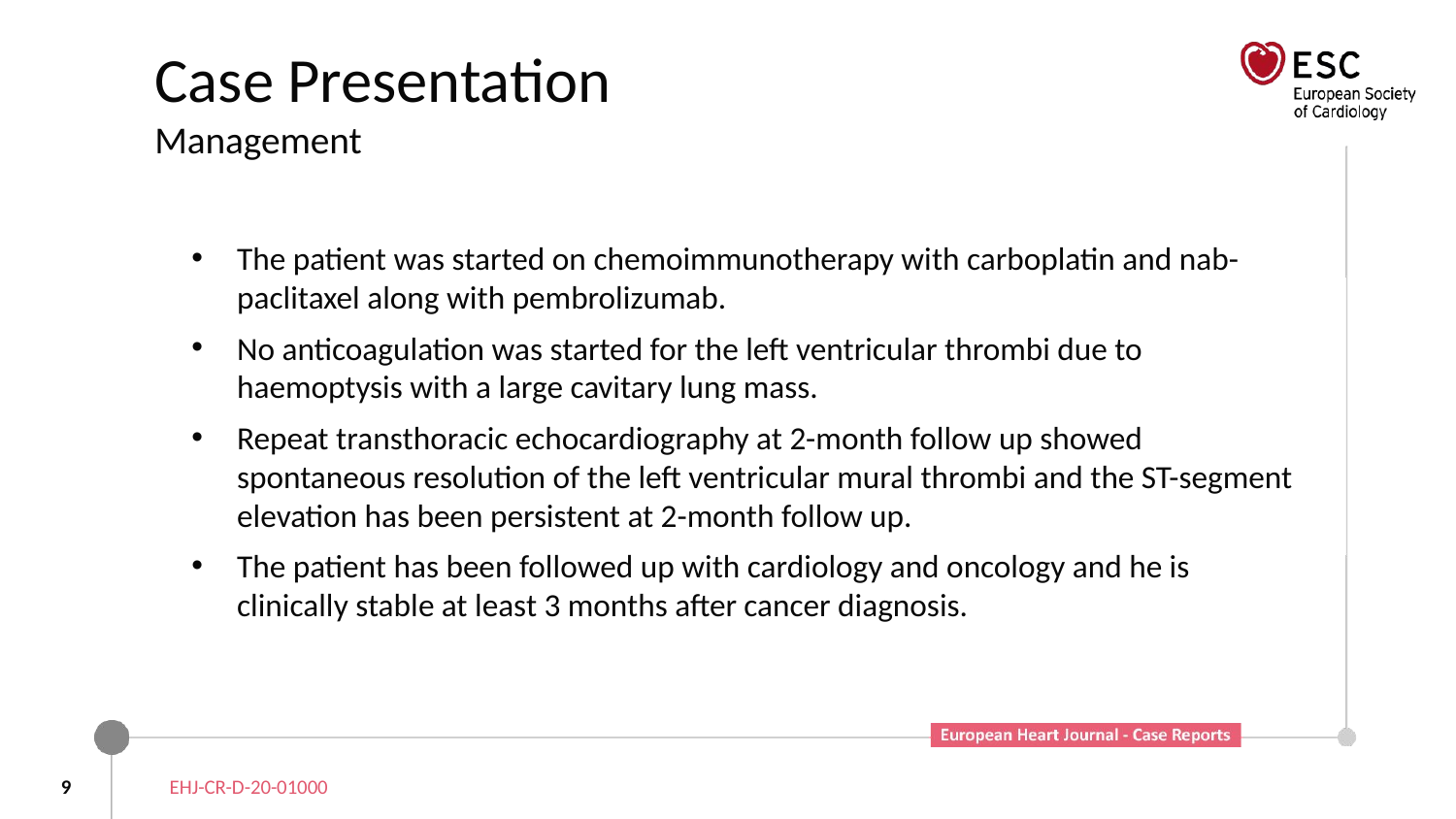

# Case Presentation Management
The patient was started on chemoimmunotherapy with carboplatin and nab-paclitaxel along with pembrolizumab.
No anticoagulation was started for the left ventricular thrombi due to haemoptysis with a large cavitary lung mass.
Repeat transthoracic echocardiography at 2-month follow up showed spontaneous resolution of the left ventricular mural thrombi and the ST-segment elevation has been persistent at 2-month follow up.
The patient has been followed up with cardiology and oncology and he is clinically stable at least 3 months after cancer diagnosis.
9
EHJ-CR-D-20-01000

## Slide 10
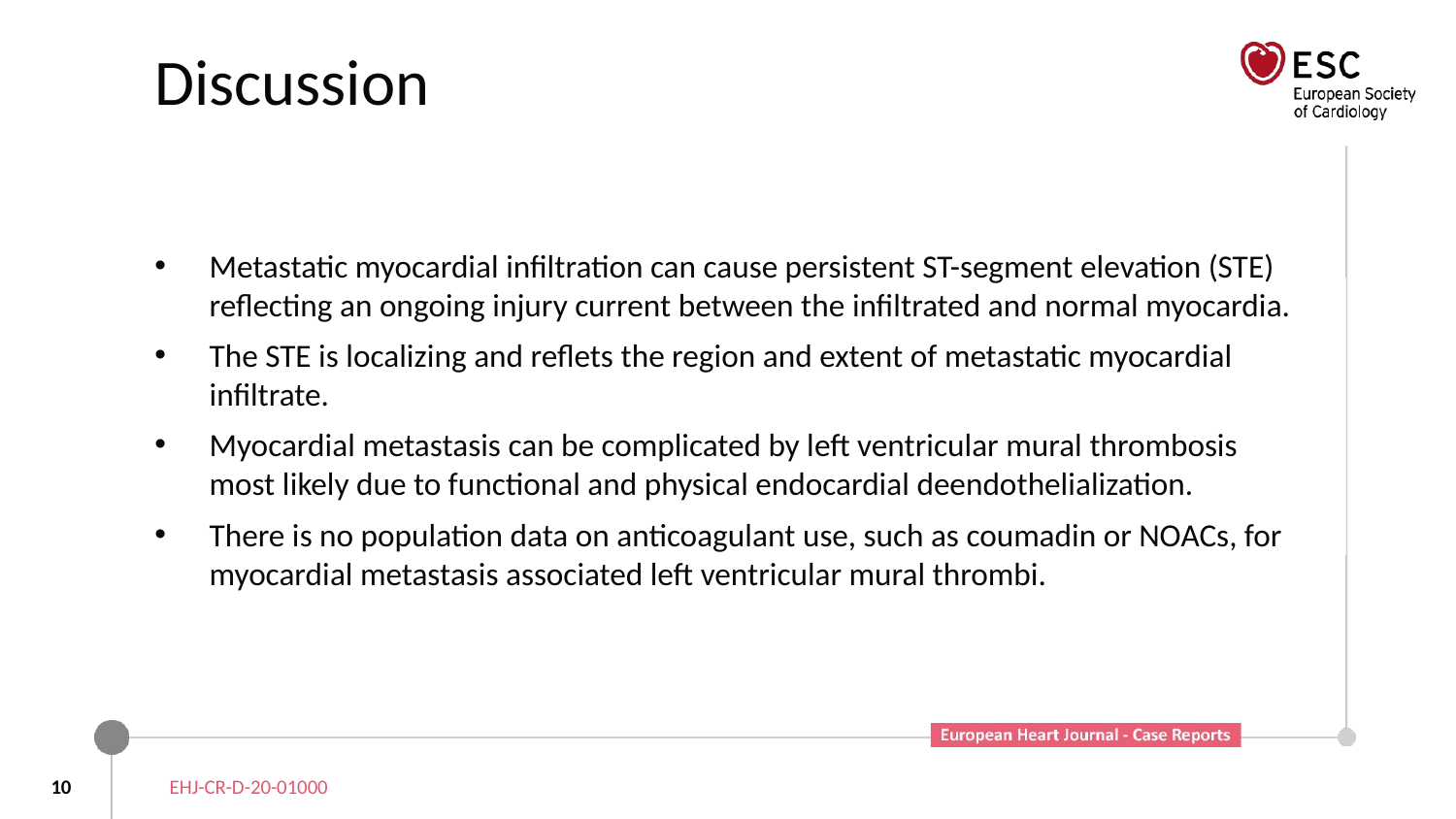

# Discussion
Metastatic myocardial infiltration can cause persistent ST-segment elevation (STE) reflecting an ongoing injury current between the infiltrated and normal myocardia.
The STE is localizing and reflets the region and extent of metastatic myocardial infiltrate.
Myocardial metastasis can be complicated by left ventricular mural thrombosis most likely due to functional and physical endocardial deendothelialization.
There is no population data on anticoagulant use, such as coumadin or NOACs, for myocardial metastasis associated left ventricular mural thrombi.
10
EHJ-CR-D-20-01000

## Slide 11
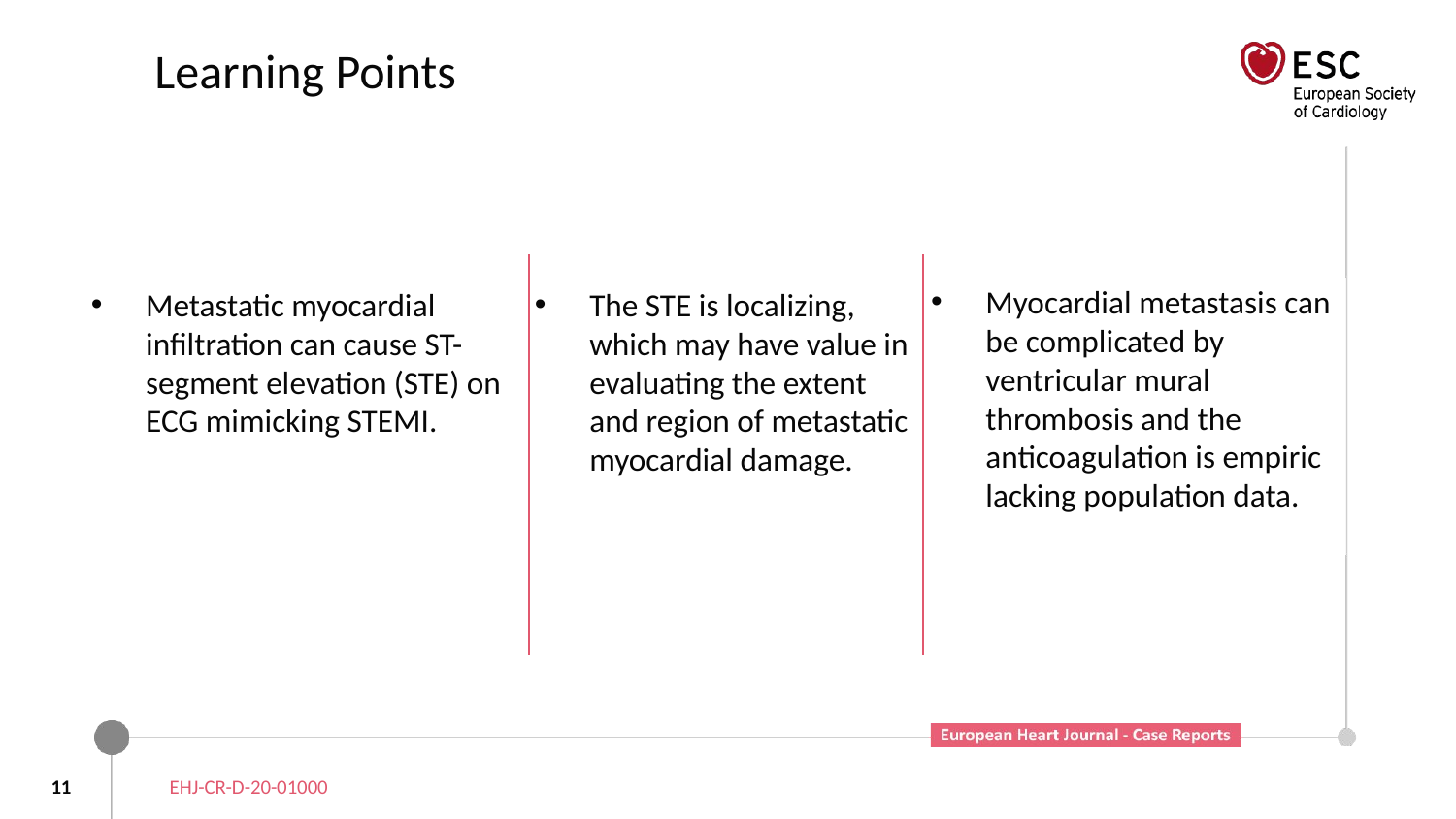

# Learning Points
Myocardial metastasis can be complicated by ventricular mural thrombosis and the anticoagulation is empiric lacking population data.
Metastatic myocardial infiltration can cause ST- segment elevation (STE) on ECG mimicking STEMI.
The STE is localizing, which may have value in evaluating the extent and region of metastatic myocardial damage.
11
EHJ-CR-D-20-01000

## Slide 12
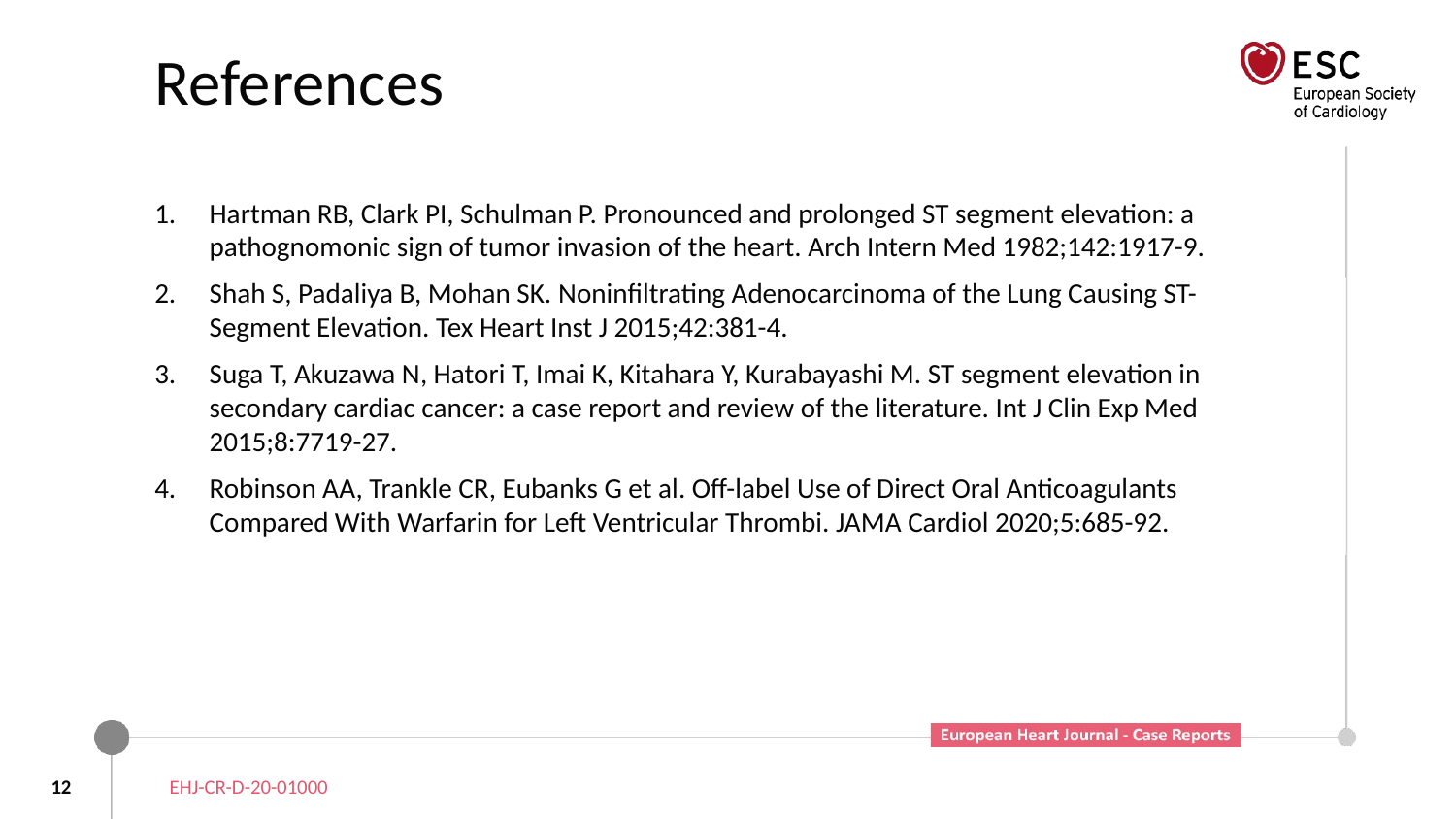

# References
Hartman RB, Clark PI, Schulman P. Pronounced and prolonged ST segment elevation: a pathognomonic sign of tumor invasion of the heart. Arch Intern Med 1982;142:1917-9.
Shah S, Padaliya B, Mohan SK. Noninfiltrating Adenocarcinoma of the Lung Causing ST-Segment Elevation. Tex Heart Inst J 2015;42:381-4.
Suga T, Akuzawa N, Hatori T, Imai K, Kitahara Y, Kurabayashi M. ST segment elevation in secondary cardiac cancer: a case report and review of the literature. Int J Clin Exp Med 2015;8:7719-27.
Robinson AA, Trankle CR, Eubanks G et al. Off-label Use of Direct Oral Anticoagulants Compared With Warfarin for Left Ventricular Thrombi. JAMA Cardiol 2020;5:685-92.
12
EHJ-CR-D-20-01000
